# Impact of the COVID-19 pandemic and the lockdown period on the number of hospitalizations for acute mania

**DOI:** 10.1192/j.eurpsy.2021.783

**Published:** 2021-08-13

**Authors:** A. Aissa, H. Ghabi, M. Ben Alaya, U. Ouali, S. Meddouri, F. Nacef

**Affiliations:** 1 Psychiatry A, Razi Hospital, Manouba, Tunisia; 2 Psychiatry A Department, Razi Hospital, Manouba, Tunisia

**Keywords:** protective factors, covid 19, acute mania

## Abstract

**Introduction:**

COVID-19 pandemic has affected social interaction and healthcare worldwide especially during the period of lockdown. As a result of this pandemic in Tunisia, the activity of hospital services and all non-emergency acts, in several specialties have been reduced. In psychiatry, such measures have not been taken. In the social zeitgeber hypothesis social rhythm disrupting life events such as eating, activity, and social patterns, may lead to the onset of manic episodes.

**Objectives:**

The objective of this study was to evaluate the impact of the COVID-19 pandemic on the frequency of acute mania in the context of bipolar disorder during the lockdown and post lockdown period compared to the same period during last year in a psychiatric department in Tunisia.

**Methods:**

We assessed the number of hospitalizations in our department for acute mania in the context of bipolar disorder during the lockdown period in our country, (from March 1st and May 30, 2020) and during the two months following it. We compared this frequency to that of the previous year during the same periods.

**Results:**

During the lockdown period, 17 patients were hospitalized for acute mania in the context of bipolar disorder. Sixty-seven patients were hospitalized in 2019 during the same period for acute mania. Nine hospitalizations for acute mania in the post lockdown period (between June and July 2020), were noted compared to 16 hospitalizations in the same period in 2019.

**Conclusions:**

Lockdown seemed to have a protective effect from affective episodes in bipolar disorder. Perceiving increased connectedness among families may explain these findings.

